# Full-length title: NRPPUR database search and in vitro analysis identify an NRPS-PKS biosynthetic gene cluster with a potential antibiotic effect

**DOI:** 10.1186/s12859-018-2479-5

**Published:** 2018-12-03

**Authors:** Shirley Fritz, Andriamiharimamy Rajaonison, Olivier Chabrol, Didier Raoult, Jean-Marc Rolain, Vicky Merhej

**Affiliations:** 1 IRD, APHM, MEPHI, IHU-Méditerranée Infection, Aix Marseille University, Marseille, France; 2 CNRS, Centrale Marseille, Aix Marseille University, I2M, Marseille, France

**Keywords:** NRPPUR database, NRPS-PKS biosynthetic gene cluster, Natural antibiotic, Genome mining, Human gut microbiota, In vitro analysis

## Abstract

**Background:**

Growing concern about the emergence of antibiotic resistance is compelling the pharmaceutical industry to search for new antimicrobial agents. The availability of genome sequences has enabled the development of computational mining as an important tool in the discovery of natural products with antibiotic effect.

**Results:**

NRPPUR (Non-Ribosomal Peptide and Polyketide Urmite) is a new bioinformatic tool that was created to detect polyketides and non-ribosomal peptide gene clusters (PKS and NRPS) in bacterial genomes using the rpsBlast program. The NRPPUR database was constructed locally by assembling all 3505 available sequences of NRPS-PKS that have been identified by in silico approaches to date, with 164 Biosynthetic Gene Clusters (BGCs) derived from the published literature that have demonstrated antimicrobial activity in vitro. The in silico analysis of 49 intestinal human bacterial genomes using the NRPPUR made it possible to identify 91 BGCs including 89 clusters that had never previously been described. On average, intestinal human bacterial genomes devote nearly 0.8% (±1.4% s.d.) of their genome to NRPS/PKS biosynthesis, with *Bacillus vallismortis*, *Streptomyces massiliensis* and *Bacillus subtilis* genomes apportioning 8.4, 3.6 and 3.15% of their genomes, respectively. When using the cross-streak method, *S. massiliensis* displayed antibacterial activity against many Gram-positive and negative bacteria including methicillin-resistant *Staphylococcus aureus* (MRSA).

**Conclusions:**

NRPPUR has proven to be a very useful tool for the primary in silico selection of species with potential antimicrobial activity and human microbiota could be the future source of new antimicrobial discoveries. Further exploration of this and other ecological niches, coupled with high-throughput antibacterial activity screening should be envisaged.

**Electronic supplementary material:**

The online version of this article (10.1186/s12859-018-2479-5) contains supplementary material, which is available to authorized users.

## Background

Antibiotics have achieved major advances in medicine and surgery, saving patients’ lives and extending the expected human lifespan [1]. Following the golden era when natural antibiotics were discovered and prescribed in 1925–1950, the chemistry era followed in the 1970s, with synthetic tweaking to improve activity. However, in 2000, the resistance era, largely due to the overuse and misuse of these medications, began [2, 3]. This coincided with the development of new technologies such as the manipulation of recombinant DNA and the high-throughput synthesis of chemicals that has given rise to hopes of drug discoveries other than antibiotics [4]. Although the high-throughput biochemical screening of large collections of syntheses has provided some interesting leads, the complexity and diversity of these molecules has been insufficient to provide the same level of bioactivity as found in naturally occurring antibiotics. It has been suggested that the coexistence of microbes with other microbes and fungi in the environment leads to selection of the most potent targets so that the best source of new antibiotics are compounds naturally produced by microorganisms [5]. It has therefore been recommended that natural products are revisited as an alternative to synthetic collections following the methods in the “golden age of the discovery of antibiotics” that screened microbial cell extracts from soil to find new antibiotic scaffolds. It has been also recommended that new technologies are embraced to overcome problems of compound discovery. Thus, the exploration of genome sequences of microorganisms and data from metagenomics of the microbial dark matter- microorganisms that have resisted to easy cultivation in the laboratory [6] has revealed a very large spectrum of potential for secondary metabolites with potential antibiotic functions [7].

Microbial secondary metabolites are organic compounds that are not directly involved in primary growth and development, but rather have auxiliary functions including defense and communication [8]. Natural antimicrobial products consist mainly of two groups i) bacteriocins [9] where biosynthesis is carried out conventionally via ribosome, and ii) polyketides (PKS) and non-ribosomal peptides (NRPS) where biosynthesis is ribosome independent. The atypical biosynthesis of NRPS and PKS known as “thiotempling” is supported by a multi-enzymatic, multi-domain synthases NRPSs and PKSs, respectively that add amino-acid monomers for NRPS and acyl Coenzyme A for PKS products. The primary sequence of the peptide product is determined by the sequential arrangement of active sites called modules within NRPSs and modular PKSs. These modules contain multiple functional domains that are necessary for catalyzing each condensation and chain elongation or modification reaction [10–12]. Genes encoding biosynthetic enzymes for the synthesis of these secondary metabolites are typically co-localized on the chromosome and are referred to as “biosynthetic gene clusters” (BGCs). Since the first elucidation of the PKS gene cluster for erythromycin in the early 1990s [13, 14], many gene clusters responsible for the biosynthesis of NRPS and PKS have been reported and deposited in International Nucleotide Sequence Database Collection (INSDC) entries (DDBJ/GenBank/EMBL) [15]. In addition, the community-driven website developed many specialized pieces of software such as Antismash [16–18] and Streptome DB [19] that enabled the detection of NRPS and PKS [20–24] in a wide range of microorganisms such as Bacteria, Fungi, Archaea and Eukarya. The general principle behind in silico mining consists of using a library of enzymes/protein domains commonly observed in secondary metabolite biosynthetic pathways to identify homologues in the genome sequences of the organisms of interest. For this task, sequence based comparison software, such as BLAST [25] or DIAMOND [26], or profile-based tools such as HMMer [27] are usually used. Together, the stunning advances in genome sequencing and informatics tools are creating the conditions necessary to support the discovery of narrow-spectrum potent antibiotics. However, large-scale gene dispensability studies using microbial gene cloning, protein expression and high-throughput screening revealed that these databases contain numerous targets that were not always bioactive when tested in vitro [28, 29].

In this paper, we present an in silico/in vitro combined strategy for identifying NRPS and PKS in the human gut microbiota. With this aim, we built an updated database, named NRPPUR (Non-Ribosomal Peptide and Polyketide Urmite), containing gene sequences for NRPS/PKS clusters, which products and corresponding extracts have demonstrated an interesting activity using antimicrobial testing methods during in vitro investigation. NRPPUR was queried to make the functional annotation using RPS-BLAST (Reverse Position-Specific Blast) in order to decipher NRPS-PKS BGCs on 49 bacterial genomes first isolated from human gut microbiota using the “culturomics” approach [30]. The antimicrobial activity of the identified producers has been tested in vitro using the cross-streak method. The combined strategy using the “culturomics- genomic-bioinformatic-antibiogram” platform has significant potential to discover new candidate antibiotic producers.

## Methods

### NRPPUR database construction

**Fig. 1:**
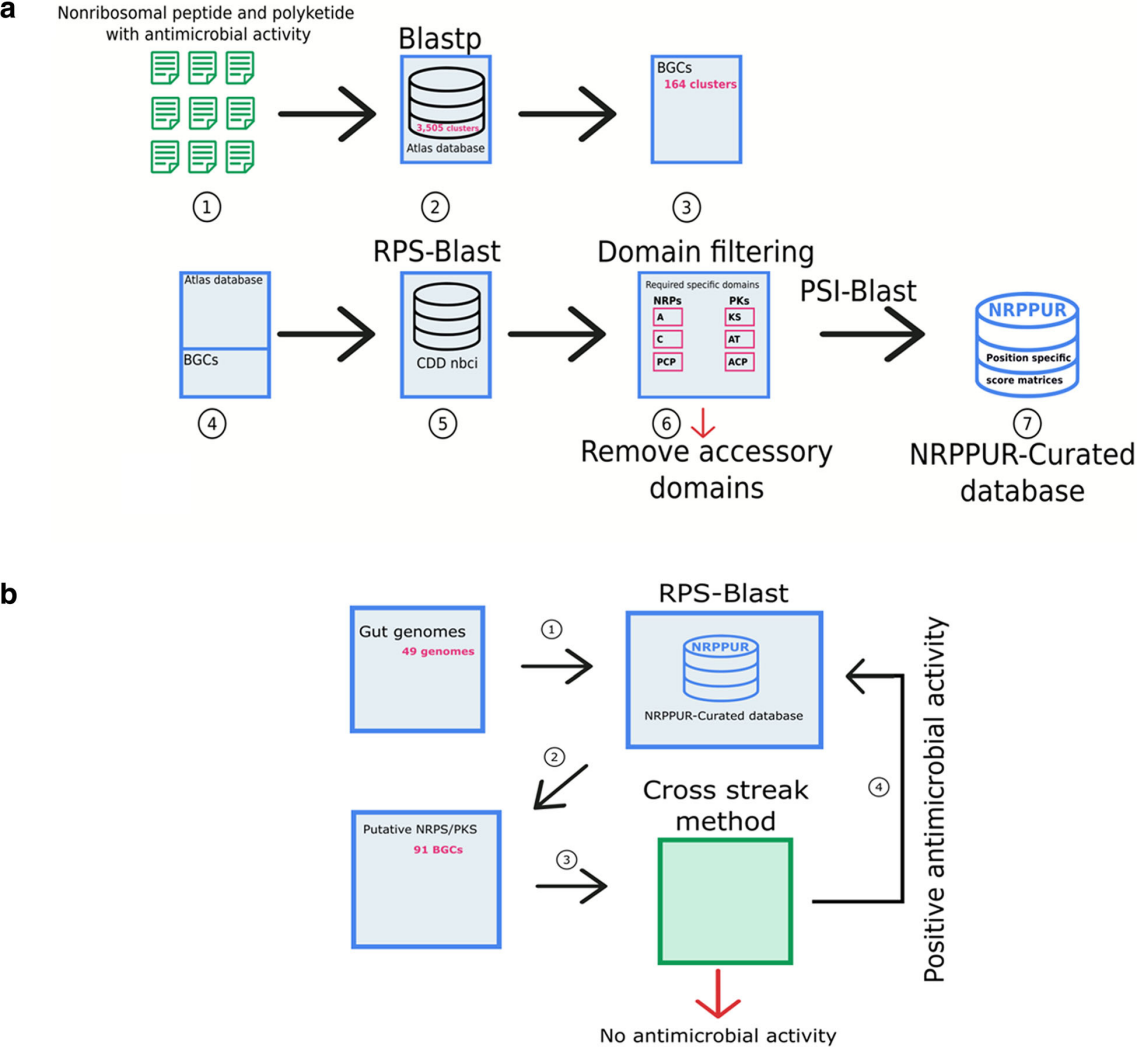
NRPPUR database construction and utilization. **a** Flowchart depicting the elaboration of NRPPUR DB. 1- Literature scan to find NRPS/PKS clusters with experimentally evidence of antimicrobial activity, 2- BLASTp analysis against Atlas database and removal of duplicate sequences, 3- Recuperation of 164 Biosynthetic Gene Clusters, 4- Assembly of the 164 BGCs to the existing Atlas database, 5- Identification of NRPS/PKS domains using RPS-BLAST against Conserved Domain Database, 6- Conservation of the minimal functional domains adenylation (A), condensation (C) and peptidyl carrier protein (PCP) in the case of NRPS and acyltransferase (A) domain, an acyl carrier protein (ACP) and a ketoacyl synthase (KS) domains in the case of PKS, 7- Elaboration of Position Specific Score Matrices for each domain using PSI-BLAST. **b** Schematic diagram representing the different steps for detection of NRPS/PKS clusters. 1- Analysis of 49 gut genomes using RPS-BLAST against NRPPUR DB, 2- Identification of putative NRPS/PKS clusters, 3- In vitro verification of antimicrobial activity using Cross-streak method, 4- Database increment with BGCs from species showing positive antimicrobial activity. The in silico steps are shown in blue and the experimental in vitro steps in green

Data collection began with a comprehensive review of the literature that reported discoveries of biosynthetic clusters encoding for secondary metabolites that have showed an antimicrobial activity in vitro*.* A literature search was conducted on PubMed, using keywords such as “NRPS”, “PKS”, “natural product biosynthesis”, “biosynthetic gene clusters” and “antimicrobial activity”. Further literature analysis was carried out using a paper recommendation system, PubMedScan (http://medals.jp/pubmedscan/), which automatically reports articles highly related to a collection of literature. The INSCD accession numbers corresponding to the BGCs were extracted from these articles and used to retrieve the corresponding nucleotide sequences from GenBank [15]. BGC sequences were annotated with the Rapid Annotation using Subsystems Technology (RAST) [31]. Protein sequences of these experimentally characterized NRPS and PKS clusters were added to the largest NRPS-PKS currently available database, Atlas database [32] to constitute the Non-Ribosomal Peptides and Polyketides URmite DataBase (NRPPUR DB). Duplicate sequences were removed with the help of BLASTp program. The non-redundant dataset of NRPS/PKS sequences was submitted to RPS-BLAST (Reverse Position-Specific Blast) search against the Conserved Domain Database (CDD) [33] in order to determine the catalytic domains of NRPS and PKS. NRPS/PKS protein domains consist of obligatory core domains for addition of each peptide and optional domains responsible for modification of the peptide backbone. The present version of NRPPUR DB was curated to contain only the main domains of the minimal module for NRPS and PKS biosynthetic systems. A minimal set of domains in a NRPS comprises an adenylation (A) domain for selection and activation of amino acid monomers, a condensation (C) domain for catalyzing the formation of peptide bonds and a peptidyl carrier protein (PCP) domain for transferring the monomers/growing chain to various catalytic sites. A minimal set of domains in a PKS comprises an acyl carrier protein (ACP) domain which is catalyzed by an acyltransferase (AT) domain and the ketoacyl synthase (KS) domain for sequential decarboxylative condensations. Sequences corresponding to each predicted domain were aligned to build a domain model’s position-specific scoring matrix (PSSM) using PSI-BLAST. Therefore, sequences were aligned via the MAFFT program (version 7.310) [34]. All PSSM files obtained were grouped and arranged in RPS database format using BLAST program (makeprofiledb) [25]. Figure 1a depicts the diagram of the construction of NRPPUR DB.

### In silico screening for antimicrobial agents

NRPPUR DB can be used to analyze protein sequences containing potential NRPS or PKS domains (Fig. 1b). Since the database contains PSSMs that have been prepared from the main domain alignments of NRPS/PKS, putative BGCs can be identified using RPS-BLAST that compares the query protein sequence against the pre-calculated PSSMs (E-value less than 0.0001). Therefore, BGCs encoding for NRPS-PKS have to present relatively adjacent genes with significant RPS-BLAST hit to two or three main domains: A, C and/or PCP domains in the case of NRPs and KS, AT and/or ACP in the case of PKs. Significant hits to at least two main domains attributed to NRPS and PKS are required for the prediction of an hybrid NRPS/PKS cluster where a single protein contains modules from both NRPS as well as PKS systems. It is noteworthy that a predicted cluster does not necessarily correspond to a single operon since the orientations of genes within the cluster may not be the same and there may be intervening genes in the cluster. In application of this approach, we have analyzed the genomes of 49 human gut bacterial strains that were isolated using culturomics. The 49 genomes corresponded to species from Firmicutes (43 species), Actinobacteria (5 species) and Proteobacteria (1 species). Of these, 27 are new species that have never previously been isolated. We developed a web interface based on the Django framework, for the identification of the main catalytic domains of NRPS/PKS.

### In vitro screening for antimicrobial activity

Based on in silico results, the genomes of microorganisms containing the largest number of NRP-PK clusters were selected for further in vitro antimicrobial assays using a modified cross-streak method [35] (Fig. 1b). This method allows assessing antagonistic property of the NRP/PK-producing bacterium against a panel of test microorganisms. Briefly, a 10^7^ CFU/mL suspension of the microbial strain of interest was seeded by a single streak in the center of the upper part of the COS agar plate, bioMerieux France and incubated at 30 °C under aerobic condition, for five days. These culture conditions depend on the bacterium of interest. The five-day incubation was done to provide enough time for the bacterium of interest to produce the presumed antibiotic substance, which will diffuse into the agar medium. Then, the plate was seeded with the test microorganisms by single streaks perpendicular to the central streak, each streak corresponding to a test microorganism. The lower part of the agar was seeded with individual streaks of the test microorganisms that were used as controls of the culture. After further incubation of 48 h at 37 °C under aerobic conditions, the antimicrobial interactions were analyzed by observing the inhibition zone size. Presence of reduced growth of test microorganism near the growth of the NRP/PK-producing bacterium was considered as positive for antagonistic activity. The test microorganisms were selected among pathogenic strains that were isolated from clinical samples in the bacteriology laboratory at the La Timone Hospital in Marseille, France. These included human pathogenic Gram-positive bacteria such as *Staphyloccocus aureus meticillin resistant, Staphylococcus aureus meticillin sensitive, Staphyloccocus epidermidis, Enterococcus faecalis* and *Bacillus cereus,* Gram-negative bacteria such as *Klebsiella pneumoniae, Escherichia coli* and *Pseudomonas aeruginosa* and yeasts such as *Candida albicans*.

## Results

### NRPPUR database

**Fig. 2:**
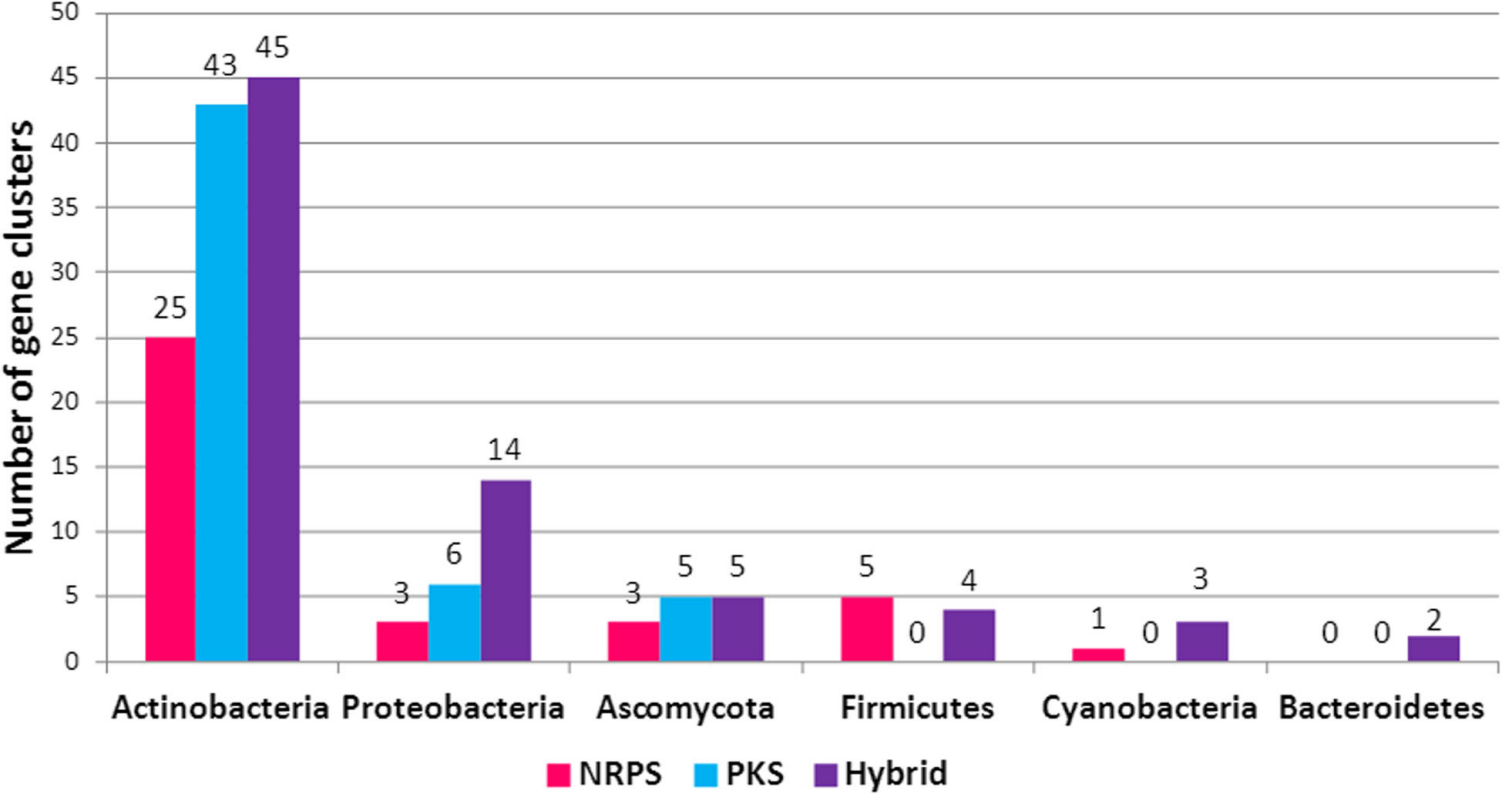
Distribution of the 164 antimicrobial NRPS-PKS and hybrid BGCs according to the phylum

The literature search resulted in 172 NRPS-PKS BGC sequences with antimicrobial secretion being extracted from Genbank. Of these, eight sequences were discarded because they didn’t show any specific hit with the conserved domains of NRPS-PKS. The other 164 sequences which were considered as NRPS-PKS BGC sequences represent the only sequences resulting from experimental data with validated antibacterial activity (Additional file 1: Table S1). Most of these BGCs, 113 (68%), were found in *Actinobacteria*, 23 in *Proteobacteria*, 13 in *Ascomycota*, nine in the *Firmicutes*, three in *Cyanobacteria* and two in *Bacteroidetes* (Fig. 2). Of these, 130 sequences showed homology within ATLAS database when using BLASTP while 34 sequences showed no homology with existing databases such as Antismash.

**Fig. 3:**
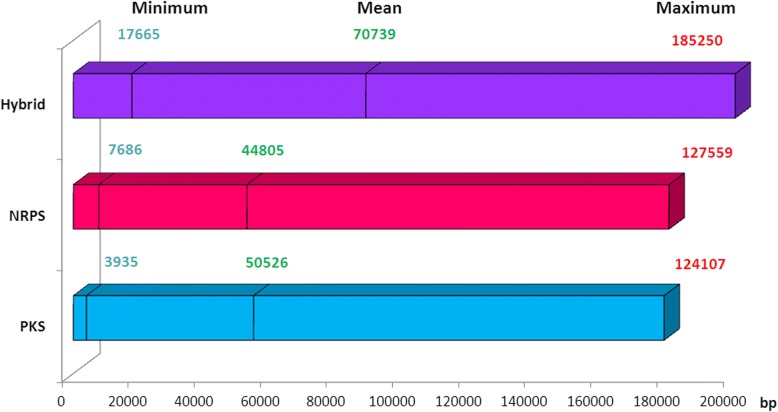
Size of the different types of gene clusters

The 164 BGCs showed great variability in size and in composition. They ranged from 3.93 to 185.25 Kbp and 155 BGCs sequences (94%) were larger than 10 Kbp. They were classified into 37 NRPS and 54 PKS, and 73 hybrid types, according to the presence of core domains of NRPS, PKS, or both systems, respectively. The mean size was 45 Kbp for NRPS, 50 Kbp for PKS and as much as 71 Kbp for hybrid BGCs (Fig. 3). The number of functional domains of these gene clusters ranged from 2 to 52 domains. The largest NRPS displayed 26 domains whereas the largest PKS had 43 domains, and the longest hybrid possessed 52 domains that constitute 1 NRPS and 9 PKS. Some clusters lacked the minimal set of domains to form modules, suggesting that some domains might be active for many modules. Thus, a high rate of mixed organization combining modular and non-modular synthase was shown in all enzyme types, especially in hybrid enzymes (14, 16 and 70% of the PKS, NRPS and hybrid enzymes had a mixed organization, respectively). PKS synthases had frequent non-modular organization whereas NRPS had modular enzyme organization (Table 1). All BGCs found in fungi had a mixed organization except one enzyme that had a modular organization. This result needs to be confirmed with more BGC from fungi. Overall, the majority of the 164 BGCs were hybrid clusters that tend to be larger and possess more functional domains with a non-modular organization, most probably giving rise to more complex products than stand-alone NRPS and PKS gene clusters.

**Table 1:** Summary of NRPS/PKS gene clusters organization and composition

	Number of BGCs		Mean size (bp)		Mean number of enzymes	
	Modular	Non modular	Modular	Non modular	Modular	Non modular
PKS	22	21	62,414	33,077	4.1	3.4
NRPS	17	7	35,307	26,988	2.1	3.1
HYBRIDE	10	7	6282	32,177	4.2	3.9

Concerning the 34 BGCs with antimicrobial activity in vitro and positive results with RPS-BLAST but no significant homology with existing databases, they were mostly found in *Streptomyces* genus (30 out of the 34). Of these, 25 were gene clusters for PKS. Interestingly, some of these BGCs support the biosynthesis of familiar antimicrobial products, including oxytetracycline, echinocandin B, chloreamphenicol, lincomycin, pristinamycin, spiramycin, streptothricin and novobiocin (Additional file 1: Table S1). These findings demonstrate that a functional enzymatic domain research strategy is more sensitive than a similarity search methods querying existing databases of putative antimicrobial BGCs. Hence, a total of 164 sequences were incremented to the Wang et al. database to form “NRPPUR DB”, a local NRPS-PKS database composed of 3505 NRPS-PKS BGC non-redundant sequences including 715 PKS, 1568 NRPS and 1220 hybrids from Bacteria, Eukarya and Archaea (respectively 3127, 373 and 3 NRPS and PKS) (Additional file 1: Figure S1). Altogether, RPS-BLAST method against NRPPUR, a database collecting BGCs products with real antimicrobial activity, can be very effective for deciphering NRPS-PKS BGCs.

### In silico screening applications

**Fig. 4:**
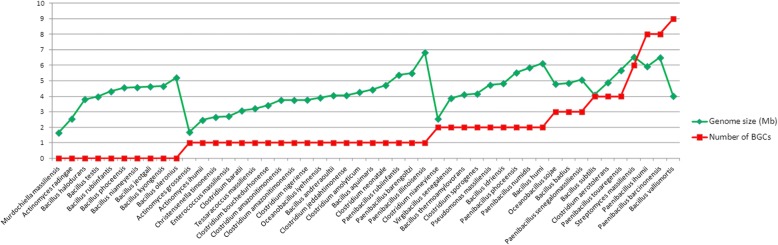
Number of BGCs found in the 49 bacterial genomes from human gut in relation with the genome-size

**Fig. 5:**
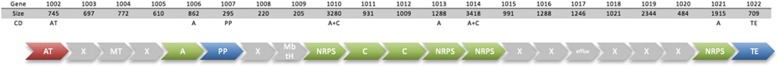
Representation of the gene cluster of *Streptomyces massiliensis.* The domains are indicated by abbreviations as acyltransferase (AT), adenylation (A), acyl carrier or peptidyl carrier domain (PP), condensation (C), thioesterase (TE)

A total of 91 BGCs were predicted from 49 intestinal human bacterial genomes using RPS-BLAST against the NRPPUR DB (Additional file 1: Table S2). Only 10 (20%) bacterial genomes studied contain no NRPS-PKS BGC. The number of predicted BGCs varied from 0 to 9 with an average of 1.86 BGCs per genome. Four genomes (*Bacillus vallismortis*, *Paenibacillus ihumii*, *Paenibacillus barcinonensis* and *Streptomyces massiliensis*) encoded the greatest total number of BGCs (9, 8, 8 and 6 respectively) of all the studied genomes. Twenty analyzed genomes showed more than one NRPS-PKS BGC. Genome size does not appear to be correlated with the number of predicted BGCs with an average of 0.53 (± 0.41 s.d.) BGCs per Mb of sequence. However, the gene clusters were nearly absent from those bacteria with a genome size less than 3.8 Mbp (Fig. 4). On average, the intestinal human bacterial genomes devote nearly 0.8% (±1.4% s.d.) of their genome to NRPS/PKS biosynthesis, with *Bacillus vallismortis*, *Streptomyces massiliensis* and *Bacillus subtilis* genomes apportioning 8.4, 3.6 and 3.15% of their genomes, respectively (Additional file 1: Table S2). The 91 BGCs were divided into 51 PKS, 19 NRPS and 21 hybrids which corresponds to a greater representation of PKS (56%) than NRPS (23%) when compared to the distribution found in the genomes of closely related species that have been studied by Wang et al. (46% NRPS, 20% PKS and 34% hybrid) (Additional file 1: Figure S2). Of these, 89 correspond to new BGCs that have never been described before. *Bacillus subtilis* genome analysis enabled the identification of already known BGCs such as *fengycin* and *surfactin* BGCs with good identification scores [36, 37]. Overall, our data mining of 49 genomes of bacteria from the intestinal microbiota showed the common distribution of BGCs encoding NRPS-PKS in bacteria from the gut. Among the genomes encoding the largest number of BGCs was the genome of a new species *Streptomyces massiliensis* from the phylum of Actinobacteria that was isolated for the first time in our laboratory from the human gut microbiota [38]. This in silico study demonstrated the presence of one NRPS, three PKS and two hybrid gene clusters, including one very large cluster showing a mixed enzymatic organization and containing all main functional domains (Fig. 5). Moreover, this cluster contains a thiosterase and an antibiotic efflux protein. Thus, likewise other bacteria producing an antibiotic *S. massiliensis* seems to have resistance genes clustered with antibiotic producing genes in accordance with a self-protection mechanism against suicide [39]. In light of these findings, we consider *S. massiliensis* to be a very promising producer strain of NRPS/PKS with effective antimicrobial potency. This newly isolated species has been chosen for further in vitro experiments for antimicrobial activity.

### In vitro antibacterial potency and spectrum of activity

**Fig. 6:**
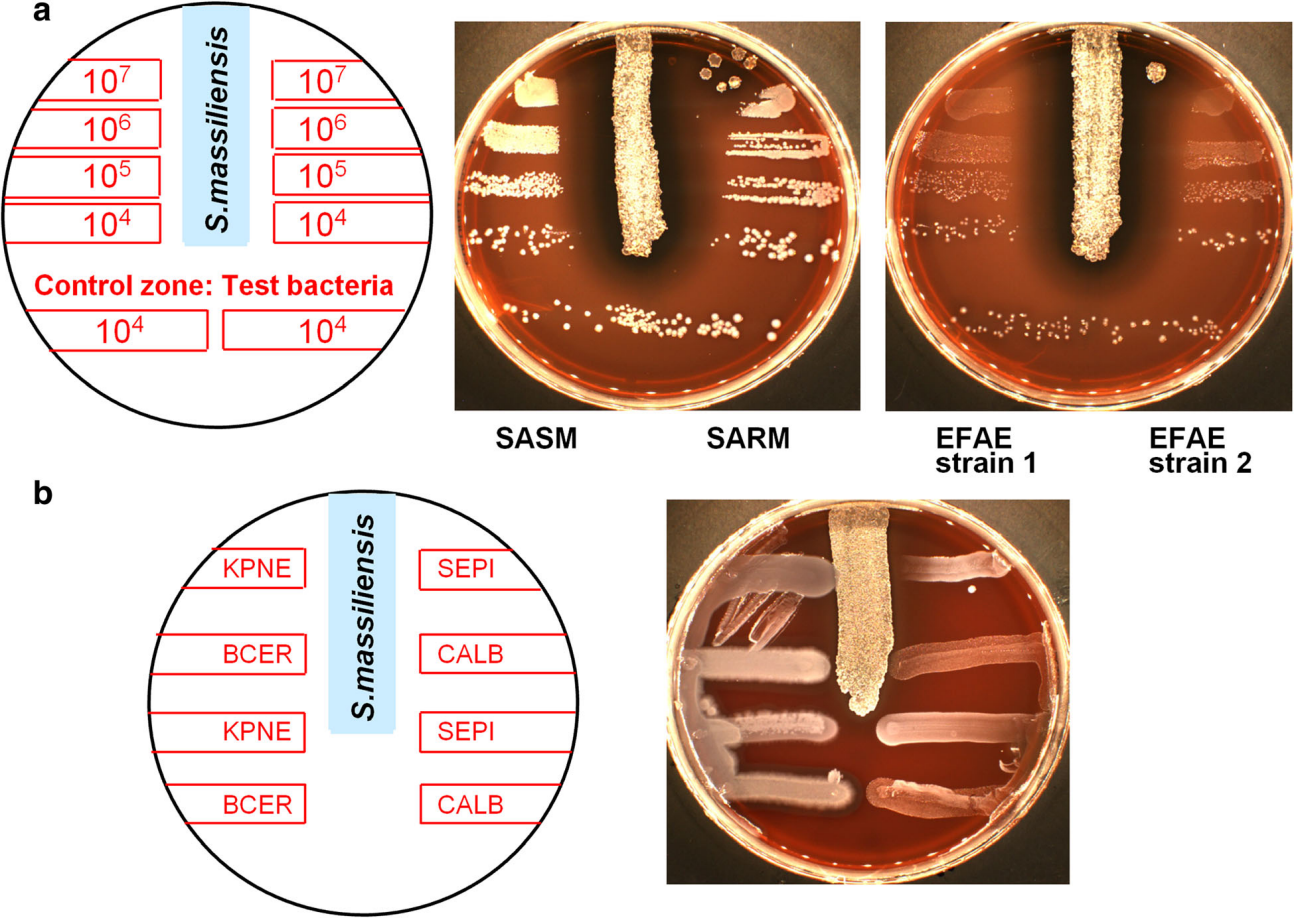
Test of antimicrobial activity of *Streptomyces massiliensis* in vitro using streak cross method*.* **a** Evaluation of the inhibitory effect of *S. massiliensis* suspensions on different concentrations of EFAE: *Enterococcus faecalis*, MRSA: methicillin-resistant *Staphylococcus aureus* and MSSA: methicillin-susceptible *Staphylococcus aureus*. Suspensions of the test bacteria at 10^4^ CFU/mL were seeded for growth control across the entire width of the lower part of the petri dish. **b** Evaluation of the inhibitory effect of *S. massiliensis* on KPNE: *Klebsiella pneumoniae;* SEPI: *Staphylococcus epidermidis;* BCER: *Bacillus cereus*; CALB: *Candida albicans*; SM: *Streptomyces massiliensis.* Two deposits were realized for the test microorganisms, one in the upper part of the agar plate, perpendicular to the streak of *S. massiliensis,* to test the inhibitory effect of *S. massiliensis* and one streak in the lower part of the agar plate to control the growth of the test microorganisms

Antimicrobial activity was checked in vitro using the cross-streak method for species containing BGCs for NRPS-PKS. *Streptomyces massiliensis* showed activity against test organisms using the cross-streak method. *S. massiliensis* displayed antibacterial activities against Gram-positive species such as methicillin-susceptible *Staphylococcus aureus* (MSSA), methicillin-resistant *S. aureus* (MRSA), *Staphylococcus epidermidis* and *Enterococcus faecalis*, except for *Bacillus cereus. S. massiliensis* was inactive against Gram-negative bacteria such as *Escherichia coli, Klebsiella pneumonia* (Fig. 6) and *Pseudomonas aeruginosa* (data not shown). *S. massiliensis* had no activity against *Candida albicans* (Fig. 6).

## Discussion

Enabled by the fast development of genome sequencing technologies, genome mining techniques looking for “natural” antimicrobial compounds are currently an important part of drug discovery efforts and many computational tools have been developed to guide wet lab experiments [20–24]. This has resulted in an increase in putative NRPS-PKS proteins predicted by gene identifications tools but for which there is no experimental evidence. Moreover, these available algorithms often propose known biosynthetic clusters similar to their own. Sequence analysis of experimentally characterized BCGs clusters seems to be a very promising strategy for identification of NRPS/PKS gene clusters [40]. Thus, NRPPUR database assembling BCGs clusters that have already demonstrated an antimicrobial activity in vitro*,* seems to be more biologically reliable than databases constructed only on the basis of bioinformatics methods. Given the difficulty of predicting antimicrobial activity in silico due to the large diversity of the protein sequences and the variable organization of NRPS/PKS clusters, our search method through the identification of functional domains seems very efficient in the detection of NRPS/PKS-producing microorganisms with antimicrobial potency as shown by our tests in vitro. Concerning the in vitro analysis, the cross-streak is an easy and relatively rapid method to investigate the antagonism between microorganisms. The high sensitivity of this method compared to other diffusion methods makes it very suitable as preliminary screening for antimicrobial activity [41]. Indeed, our searchable comprehensive database using RPS-BLAST enabled an initial in silico selection of species with potential antimicrobial activity. This represents a great gain in terms of time and money and a powerful way of selecting species that show antibacterial activity in vitro, including against highly resistant bacteria such as MRSA. NRPPUR DB provides a curated set of domain sequences of known biosynthesis cluster enzymes which enables users to judge the novelty of their sequences searches. Moreover, this collection of PKS and NRPS genes corresponding to known bioactive compounds would enable the determination of key structure-activity relationships specifically with antimicrobial activity.

The in silico analysis of genome sequences from the gut microbiota, including sequences of newly isolated fastidious species, enabled the identification of a high number of NRPS-PKS and revealed the wide spread of putative antimicrobial agents in bacteria from the human gut. Of the 49 species studied from gut microbiota, 39 genomes contained at least one NRPS-PKS (79.6%), which is higher than the proportion described in the study by Wang et al. that found only 32.3% (960/2976) of organisms studied from different environments. Human gut microbiota seems to have atypical distribution into NRPS, PKS and hybrids with a predominance of PKS. Of the newly identified BCGs, 89 clusters were deciphered for the first time by our in silico approach; they include 52 PKS, 17 NRPS, and 20 hybrids. Taken together, this work has made it possible to study the diversity and distribution of secondary metabolites in a specific environment, the human gut, which opens up the possibility of learning more about the impact of these compounds on shaping environmental habitats. Thus human gut microbiota seems to be a competitive environment [42] where NRP-PK are produced at a high rate and which may constitute one of the defensive mechanisms used by microorganisms to survive [43, 44]. Indeed, while most natural products were isolated from environmental microbial strains recently, *Staphylococcus lugdunensis,* a human commensal, was described to have a protective role against *Staphylococcus aureus* colonization in the nasal human microbiota. This may be mediated by the antibacterial non-ribosomal peptide, Lugdunin [45]. Thus, human microbiota could be the future source of new antimicrobial discoveries, and further exploration of this ecological niche, coupled with newer technologies such as cell-free assays and high-throughput screening, should be envisaged. Further transcriptomic and gene silencing approaches can confirm the implication of NRPS/PKS clusters in the observed antimicrobial potency. Moreover, our results showed that *Bacillus, Paenibacillus* and *Streptomyces* genomes were outlier in the number of NRPS/PKS clusters. While *Streptomyces* species are known to be prolific producers of antibiotics and other natural products, the high rate of NRPS/PKS in *Bacillus* spp. is likely to reflect their abundance in the microbiota and their particular ecological role involving multiple interactions with cohabiting microbes. Given that, these species should be vigorously pursued for new antimicrobial product discoveries.

## Conclusions

This work is a pioneering study to search for new NRPS-PKS naturally produced by the human digestive microbiota and showing potent antibiotic activity in vitro. The NRPPUR database integrates the latest experimentally verified information and provides standardized domain descriptions related to the gene clusters. Our database serves as a useful reference to facilitate research and development related to secondary metabolite types NRPS and PKS with potential antibiotic activity. A web interface (http://www.mediterranee-infection.com/article.php?laref=955&titre=nrppur-database-) has been developed allowing rpsBlast analyses to be performed to search for NRPS-PKS.

## Additional file

Additional file 1: **Figure S1.** A Venn diagram of PKS, NRPS, and hybrid gene-cluster numbers in NRPUR database. The gene-cluster numbers of the total, bacteria, eukarya and archaea are shown in black, red, green, and grey, respectively. **Table S1.** The 164 NRPS-PKS BGC sequences identified from the literature search resulting from experimental data with validated antibacterial activity. **Table S2.** Summary of NRPS and PKS gene clusters found in the genomes of bacteria from the gut. **Figure S2.** Distribution of NRPS, PKS, and hybrid gene clusters in bacteria from the gut a) in all studied bacteria, b) in the studied phyla (PDF 313 kb)

## Abbreviations

ACP: Acyl Carrier Protein
A-domain: Adenylation domain
AT: Acyltransferase
BGCs: Biosynthetic Gene Clusters
C-domain: Condensation domain
KS: Ketosynthase
MRSA: Methicillin-Resistant *Staphylococcus aureus*
NRPPUR: Non-Ribosomal Peptide and Polyketide Urmite
NRPS: Non-Ribosomal Peptide gene clusters
NRPs: Non-Ribosomal Peptides
PCP-domain: Peptidyl Carrier domain
PKs: Polyketides
PKS: Polyketides gene clusters
RAST: Rapid Annotation using Subsystems Technology
RPS-BLAST: Reverse Position-Specific Blast

## References

[1] Gould IM, Bal AM (2013). New antibiotic agents in the pipeline and how they can overcome microbial resistance. Virulence.

[2] Boucher HW, Talbot GH, Bradley JS, Edwards JE, Gilbert D, Rice LB (2009). Bad bugs, no drugs: no ESKAPE! An update from the Infectious Diseases Society of America. Clin Infect Dis Off Publ Infect Dis Soc Am.

[3] Alekshun MN, Levy SB (2007). Molecular mechanisms of antibacterial multidrug resistance. Cell.

[4] Brown ED, Wright GD (2016). Antibacterial drug discovery in the resistance era. Nature.

[5] Lewis K (2012). Antibiotics: Recover the lost art of drug discovery. Nature.

[6] Lock C (2015). Mining the microbial dark matter. Nature.

[7] Drissi F, Buffet S, Raoult D, Merhej V (2015). Common occurrence of antibacterial agents in human intestinal microbiota. Front Microbiol.

[8] Schwarzer D, Finking R, Marahiel MA (2003). Nonribosomal peptides: from genes to products. Nat Prod Rep.

[9] Ahmad V, Khan MS, Jamal QMS, Alzohairy MA, Al Karaawi MA, Siddiqui MU. Antimicrobial potential of bacteriocins: in therapy, agriculture and food preservation. Int J Antimicrob Agents. 2016. 10.1016/j.ijantimicag.2016.08.016 27773497

[10] Weissman KJ (2014). The structural biology of biosynthetic megaenzymes. Nat Chem biol. Sep.

[11] Jenke-Kodama H, Dittmann E (2009). Bioinformatic perspectives on NRPS/PKS megasynthases: advances and challenges. Nat Prod Rep.

[12] Khosla C, Kapur S, Cane DE (2009). Revisiting the modularity of modular polyketide synthases. Curr Opin Chem Biol.

[13] Cortes J, Haydock SF, Roberts GA, Bevitt DJ, Leadlay PF (1990). An unusually large multifunctional polypeptide in the erythromycin-producing polyketide synthase of Saccharopolyspora erythraea. Nature.

[14] Donadio S, Staver MJ, McAlpine JB, Swanson SJ, Katz L (1991). Modular organization of genes required for complex polyketide biosynthesis. Science.

[15] Benson DA, Karsch-Mizrachi I, Clark K, Lipman DJ, Ostell J, Sayers EW (2012). GenBank. Nucleic Acids Res.

[16] Medema MH, Blin K, Cimermancic P, de Jager V, Zakrzewski P, Fischbach MA, Weber T, Takano E, Breitling R (2011). AntiSMASH: rapid identification, annotation and analysis of secondary metabolite biosynthesis gene clusters in bacterial and fungal genome sequences. Nucleic Acids Res.

[17] Blin K, Medema MH, Kazempour D, Fischbach MA, Breitling R, Takano E, Weber T (2013). Nucleic Acids Res.

[18] Weber T, Blin K, Duddela S, Krug D, Kim HU, Bruccoleri R, Lee SY, Fischbach M, Müller R, Wohlleben W, Breitling R, Takano E, Medema MH (2015). Nucleic Acids Res.

[19] Lucas X, Senger C, Erxleben A, Grüning BA, Döring K, Mosch J (2013). StreptomeDB: a resource for natural compounds isolated from Streptomyces species. Nucleic Acids Res.

[20] Starcevic A, Zucko J, Simunkovic J, Long PF, Cullum J, Hranueli D (2008). ClustScan: an integrated program package for the semi-automatic annotation of modular biosynthetic gene clusters and in silico prediction of novel chemical structures. Nucleic Acids Res.

[21] Weber T, Rausch C, Lopez P, Hoof I, Gaykova V, Huson DH, Wohlleben W (2009). CLUSEAN: a computer-based framework for the automated analysis of bacterial secondary metabolite biosynthetic gene clusters. J Biotechnol.

[22] Conway KR, Boddy CN (2013). ClusterMine360: a database of microbial PKS/NRPS biosynthesis. Nucleic Acids Res.

[23] Ichikawa N, Sasagawa M, Yamamoto M, Komaki H, Yoshida Y, Yamazaki S, Fujita N (2013). DoBISCUIT: a database of secondary metabolite biosynthetic gene clusters. Nucleic Acids Res.

[24] Skinnider MA, Dejong CA, Rees PN, Johnston CW, Li H, Webster AL, Wyatt MA, Magarvey NA (2015). Genomes to natural products PRediction informatics for secondary Metabolomes (PRISM). Nucleic Acids Res.

[25] Altschul SF, Gish W, Miller W, Myers EW, Lipman DJ (1990). Basic local alignment search tool. J Mol Biol.

[26] Buchfink B, Xie C, Huson DH (2015). Fast and sensitive protein alignment using DIAMOND. Nat Methods.

[27] Finn RD, Clements J, Eddy SR (2011). HMMER web server: interactive sequence similarity searching. Nucleic Acids Res.

[28] Buysse JM (2001). The role of genomics in antibacterial target discovery. Curr Med Chem.

[29] Selzer PM, Brutsche S, Wiesner P, Schmid P, Müllner H (2000). Target-based drug discovery for the development of novel antiinfectives. Int J Med Microbiol.

[30] Lagier JC, Khelaifia S, Alou MT, Ndongo S, Dione N, Hugon P (2016). Culture of previously uncultured members of the human gut microbiota by culturomics. Nat Microbiol.

[31] Aziz RK, Bartels D, Best AA, DeJongh M, Disz T, Edwards RA, Formsma K (2008). The RAST server: rapid annotations using subsystems technology. BMC Genomics.

[32] Wang H, Fewer DP, Holm L, Rouhiainen L, Sivonen K (2014). Atlas of nonribosomal peptide and polyketide biosynthetic pathways reveals common occurrence of nonmodular enzymes. Proc Natl Acad Sci U S A.

[33] Marchler-Bauer A, Bo Y, Han L, He J, Lanczycki CJ, Lu S (2017). CDD/SPARCLE: functional classification of proteins via subfamily domain architectures. Nucleic Acids Res.

[34] Katoh K, Standley DM (2013). MAFFT multiple sequence alignment software version 7: improvements in performance and usability. Mol Biol Evol.

[35] Williston EH, Zia-Walrath P, Youmans GP (1947). Plate methods for testing antibiotic activity of actinomycetes against virulent human type tubercle bacilli. J Bacteriol.

[36] Lin TP, Chen CL, Chang LK, Tschen JS, Liu ST (1999). Functional and transcriptional analyses of a fengycin synthetase gene, fenC, from Bacillus subtilis. J Bacteriol.

[37] Nakano MM, Magnuson R, Myers A, Curry J, Grossman AD, Zuber P (1991). srfA is an operon required for surfactin production, competence development, and efficient sporulation in Bacillus subtilis. J Bacteriol.

[38] Pfleiderer A, Lagier JC, Armougom F, Robert C, Vialettes B, Raoult D (2013). Culturomics identified 11 new bacterial species from a single anorexia nervosa stool sample. Eur J Clin Microbiol Infect Dis.

[39] Wright GD (2007). The antibiotic resistome: the nexus of chemical and genetic diversity. Nat Rev Microbiol..

[40] Ansari MZ, Yadav G, Gokhale RS, Mohanty D (2004). NRPS-PKS: a knowledge-based resource for analysis of NRPS/PKS megasynthases. Nucleic Acids Res.

[41] Lertcanawanichakul M, Sawangnop S (2008). A comparison of Two Methods used for measuring the antagonistic activity of Bacillus Species. Walailak J Sci Tech.

[42] Ley RE, Peterson DA, Gordon JI (2006). Ecological and evolutionary forces shaping microbial diversity in the human intestine. Cell.

[43] Hibbing ME, Fuqua C, Parsek MR, Peterson SB (2010). Bacterial competition: surviving and thriving in the microbial jungle. Nat Rev Microbiol.

[44] Prasad S, Manasa P, Buddhi S, Singh SM, Shivaji S (2011). Antagonistic interaction networks among bacteria from a cold soil environment. FEMS Microbiol Ecol.

[45] Zipperer A, Konnerth MC, Laux C, Berscheid A, Janek D, Weidenmaier C (2016). Human commensals producing a novel antibiotic impair pathogen colonization. Nature.

