# Supplementary material for: Full-length title: NRPPUR database search and in vitro analysis identify an NRPS-PKS biosynthetic gene cluster with a potential antibiotic effect
Source: BMC Bioinformatics. 2018 Dec 3;19:463. doi: 10.1186/s12859-018-2479-5 (PMC6276269; doi:10.1186/s12859-018-2479-5)

Additional Figure 1: A Venn diagram of PKS, NRPS, and hybrid gene-cluster numbers in NRPUR database. The gene-cluster numbers of the total, bacteria, eukarya and archaea are shown in black, red, green, and grey, respectively.

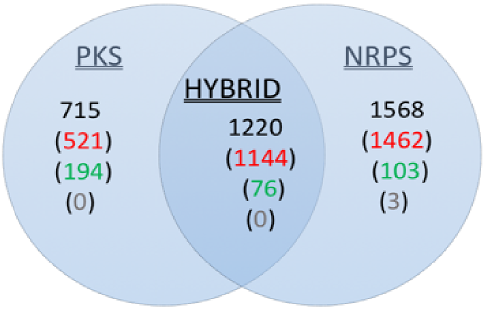

Additional table 1: The 164 NRPS-PKS BGC sequences identified from the literature search resulting from experimental data with validated antibacterial activity

Antibiotic name	BGC type	Microbial strain	Phylum	Length (bp)	Accession	PMID
Echinocandin B	NRPS	Emericella rugulosa strain NRRL 11440	Ascomycota (Fungi)	53291	JX421684	22998630
Verlamelin	NRPS	Lecanicillium sp. HF627	Ascomycota (Fungi)	26762	AB862312	24848421
Paraherquamide	NRPS	Penicillium fellutanum strain ATCC 20841	Ascomycota (Fungi)	47884	JQ708195	23213353
A40926	NRPS	Actinomadura sp. ATCC 39727	Actinobacteria (Bacteria)	89153	AJ561198	12837387
Friulimicin	NRPS	Actinoplanes friuliensis	Actinobacteria (Bacteria)	70820	AJ488769	15942003
Bacitracin	NRPS	Bacillus licheniformis	Firmicutes (Bacteria)	48774	AF007865	9427658
Fengycin	NRPS	Bacillus subtilis	Firmicutes (Bacteria)	7774	AJ011849	10438779
Gramicidin	NRPS	Brevibacillus brevis	Firmicutes (Bacteria)	13828	X61658	1560782
Tyrocidine	NRPS	Brevibacillus brevis	Firmicutes (Bacteria)	40897	AF004835	9352938
Teixobactin	NRPS	Eleftheria terrae	Proteobacteria (Bacteria)	52035	749800189	25561178
alpha-aminoadipyl-cysteinyl-valine synthetase	NRPS	Lysobacter lactamgenus	Proteobacteria (Bacteria)	11401	D50308	8737573
Aeruginosin	NRPS	Microcystis aeruginosa NIES-98	Cyanobacteria (Bacteria)	25401	FJ609416	19201978
Colistin (Polymyxin E1 & E2)	NRPS	Paenibacillus alvei	Firmicutes (Bacteria)	41172	KP262070	25609230
Massetolide A	NRPS	Pseudomonas fluorescens	Actinobacteria (Bacteria)	18770	EU199080	17241198
Massetolide A	NRPS	Pseudomonas fluorescens	Proteobacteria (Bacteria)	41109	EU199081	17241198
Dithiolopyrrolone	NRPS	Saccharothrix algeriensis strain NRRL B-24137	Actinobacteria (Bacteria)	41334	KM114209	25353334
Actinomycin	NRPS	Streptomyces anulatus ATCC 11523	Actinobacteria (Bacteria)	66086	HM038106	10212227
Telomycin	NRPS	Streptomyces canus	Actinobacteria (Bacteria)	85193	KP756960	26043159
Thienamycin	NRPS	Streptomyces cattleya	Actinobacteria (Bacteria)	32329	AJ421798	12725858
Pacidamycin	NRPS	Streptomyces coeruleorubidus strain NRRL 18370	Actinobacteria (Bacteria)	30311	HM855229	20826445
Daptomycin	NRPS	Streptomyces filamentosus	Actinobacteria (Bacteria)	127559	AY787762	15870461
A54145	NRPS	Streptomyces fradiae strain NRRL18158	Actinobacteria (Bacteria)	126933	DQ118863	16208464
Enduracidin	NRPS	Streptomyces fungicidicus	Actinobacteria (Bacteria)	83472	DQ403252	17005978
Capuramycin	NRPS	Streptomyces griseus	Actinobacteria (Bacteria)	65257	AB476988	19478828
Complestatin	NRPS	Streptomyces lavendulae	Actinobacteria (Bacteria)	55972	AF386507	11447274
Streptothricin	NRPS	Streptomyces lavendulae subsp. lavendulae	Actinobacteria (Bacteria)	34315	AB684620	22820420
Lincomycin	NRPS	Streptomyces lincolnensis	Actinobacteria (Bacteria)	38217	EU124663	19085073
Novobiocin	NRPS	Streptomyces niveus strain NCIMB 9219	Actinobacteria (Bacteria)	28076	AF170880	10770754
Pristinamycin	NRPS	Streptomyces pristinaespiralis	Actinobacteria (Bacteria)	14547	FN563143	21146568
Pristinamycin	NRPS	Streptomyces pristinaespiralis	Actinobacteria (Bacteria)	7686	FN563144	21146568
Pristinamycin	NRPS	Streptomyces pristinaespiralis	Actinobacteria (Bacteria)	22449	X98690	9006024
Streptothricin	NRPS	Streptomyces rochei	Actinobacteria (Bacteria)	34051	AB684619	22820420
Napsamycin	NRPS	Streptomyces sp. DSM 5940	Actinobacteria (Bacteria)	35298	HQ287563	21290549

Antibiotic name	BGC type	Microbial strain	Phylum	Length (bp)	Accession	PMID
Arylomycin	NRPS	Streptomyces sp. Tue 6075	Actinobacteria (Bacteria)	57630	827541682	22941370
Valinomycin	NRPS	Streptomyces tsusimaensis ATCC 15141	Actinobacteria (Bacteria)	39345	76884858	16511823
Chloremphenicol	NRPS	Streptomyces venezuelae ATCC 10712	Actinobacteria (Bacteria)	14159	AF262220	11577160
Viomycin	NRPS	Streptomyces vinaceus strain ATCC 11861	Actinobacteria (Bacteria)	36401	32967975	12936980
Bikaverin	PKS	Gibberella fujikuroi	Ascomycota (Fungi)	7783	AJ278141	20376635
Citrinin	PKS	Monascus purpureus	Ascomycota (Fungi)	9269	AB167465	16000748
Griseofulvin	PKS	Penicillium aethiopicum strain IBT 5753	Ascomycota (Fungi)	55000	KM434884	20534346
Radicicol	PKS	Pochonia chlamydosporia strain ATCC 16683	Ascomycota (Fungi)	31332	EU520419	18567690
Hypocrellin	PKS	Shiraia sp. slf14	Ascomycota (Fungi)	50000	23574639	24503982
Pradimicin	PKS	Actinomadura hibisca strain P157-2	Actinobacteria (Bacteria)	37100	EF151801	18051306
Esperamicin	PKS	Actinomadura verrucosospora strain ATCC 39334	Actinobacteria (Bacteria)	6689	AY267372	14528002
Quartromicin	PKS	Amycolatopsis orientalis	Actinobacteria (Bacteria)	82338	JF970188	23102224
Bongkrekic acid	PKS	Burkholderia gladioli strain DMSZ11318	Proteobacteria (Bacteria)	67546	JX173632	Unpublished
Gephyronic acid	PKS	Cystobacter violaceus	Proteobacteria (Bacteria)	63402	KF479198	24298873
Tetrocarcin A	PKS	Micromonospora chalcea	Actinobacteria (Bacteria)	108236	EU443633	18586939
Calicheamicin	PKS	Micromonospora echinospora	Actinobacteria (Bacteria)	90348	AF497482	12183629
Megalomicin	PKS	Micromonospora megalomicea subsp. nigra	Actinobacteria (Bacteria)	47981	AF263245	10972798
Etnangien	PKS	myxobacterium Sorangium	Proteobacteria (Bacteria)	120001	AM746676	12420170
Jerangolid	PKS	Polyangium cellulosum	Proteobacteria (Bacteria)	67323	DQ897668	17185223
Soraphen	PKS	Sorangium cellulosum	Proteobacteria (Bacteria)	67523	U24241	7601830
Sorangicin	PKS	Sorangium cellulosum strain So ce12	Proteobacteria (Bacteria)	124107	HM584908	20715267
Spiramycin	PKS	Streptomyces ambofaciens	Actinobacteria (Bacteria)	11609	AM709783	18048924
Spiramycin	PKS	Streptomyces ambofaciens	Actinobacteria (Bacteria)	41674	AM709784	18048924
Lactimidomycin	PKS	Streptomyces amphibiosporus	Actinobacteria (Bacteria)	50543	GQ274954	25405956
Chlorothricin	PKS	Streptomyces antibioticus	Actinobacteria (Bacteria)	101867	DQ116941	16793515
Oleandomycin	PKS	Streptomyces antibioticus	Actinobacteria (Bacteria)	24568	AF220951	10908114
Chalcomycin	PKS	Streptomyces bikiniensis	Actinobacteria (Bacteria)	85915	AY509120	15561847
Niddamycin	PKS	Streptomyces caelestis.	Actinobacteria (Bacteria)	41097	AF016585	9393718
Chartreusin	PKS	Streptomyces chartreusis	Actinobacteria (Bacteria)	19580	AJ786382	15911378
Urdamycin	PKS	Streptomyces fradiae	Actinobacteria (Bacteria)	5700	X87093	7592377
FD-891	PKS	Streptomyces graminofaciens	Actinobacteria (Bacteria)	74321	AB469193	20589823
Gilvocarcin	PKS	Streptomyces griseoflavus	Actinobacteria (Bacteria)	33846	AY233211	12822997
Fredericamycin	PKS	Streptomyces griseus	Actinobacteria (Bacteria)	33064	AF525490	16305230
Griseusin	PKS	Streptomyces griseus	Actinobacteria (Bacteria)	5364	X77865	8169211
Halstoctacosanolide	PKS	Streptomyces halstedii	Actinobacteria (Bacteria)	107687	AB241068	16568718
lasalocid	PKS	Streptomyces lasaliensis	Actinobacteria (Bacteria)	3935	FM173265	Unpublished
Lasalocid	PKS	Streptomyces lasaliensis	Actinobacteria (Bacteria)	81959	AB449340	19129623
Amphotericine B	PKS	Streptomyces nodosus	Actinobacteria (Bacteria)	113193	AF357202	11451671

Antibiotic name	BGC type	Microbial strain	Phylum	Length (bp)	Accession	PMID
Nogalamycin	PKS	Streptomyces nogalater	Actinobacteria (Bacteria)	19632	AJ224512	8668120
Nystatin	PKS	Streptomyces noursei ATCC 11455	Actinobacteria (Bacteria)	123580	AF263912	10873841
Elloramycin	PKS	Streptomyces olivaceus	Actinobacteria (Bacteria)	24201	AM900040	18310024
Borrelidin	PKS	Streptomyces parvulus	Actinobacteria (Bacteria)	74787	AJ580915	15112998
Piericidin	PKS	Streptomyces piomogenus	Actinobacteria (Bacteria)	49420	HQ840721	22365607
Resistomycin	PKS	Streptomyces resistomycificu	Actinobacteria (Bacteria)	16662	AJ585192	14982421
Frenolicin	PKS	Streptomyces roseofulvus	Actinobacteria (Bacteria)	25306	AF058302	8181754
Griseorhodin A	PKS	Streptomyces sp.	Actinobacteria (Bacteria)	36905	AF509565	12323376
Benastatin	PKS	Streptomyces sp. A2991200	Actinobacteria (Bacteria)	17480	AM501485	17439117
Medermycin	PKS	Streptomyces sp. AM-7161	Actinobacteria (Bacteria)	36202	AB103463	12855716
Alnumycin	PKS	Streptomyces sp. CM020	Actinobacteria (Bacteria)	31030	EU852062	18940666
FD-594	PKS	Streptomyces sp. TA-0256	Actinobacteria (Bacteria)	43026	AB469194	9589064
Lysolipin	PKS	Streptomyces tendae	Actinobacteria (Bacteria)	43202	AM492533	20399259
Jadomycin B	PKS	Streptomyces venezuelae	Actinobacteria (Bacteria)	7064	AF126429	7881555
Methymycin and Pikromycin	PKS	Streptomyces venezuelae	Actinobacteria (Bacteria)	37948	BD217173	Unpublished
Pikromycin	PKS	Streptomyces venezuelae	Actinobacteria (Bacteria)	37948	AF079138	9770448
Granaticin	PKS	Streptomyces vietnamensis	Actinobacteria (Bacteria)	37480	GU233672	21732034
Granaticin	PKS	Streptomyces violaceoruber	Actinobacteria (Bacteria)	39250	AJ011500	9831526
Nigericin	PKS	Streptomyces violaceusniger	Actinobacteria (Bacteria)	95700	DQ354110	17584617
Avilamycin	PKS	Streptomyces viridochromogenes	Actinobacteria (Bacteria)	59816	AF333038	8768522
Equisetin	Hybrid	Fusarium heterosporum	Ascomycota (Fungi)	60504	AY700570	26160390
Equisetin	Hybrid	Fusarium heterosporum strain ATCC 74349	Ascomycota (Fungi)	41299	187940966	15724180
Hypothemycin	Hybrid	Hypomyces subiculosus	Ascomycota (Fungi)	36275	EU520417	18567690
Viridicatumtoxin	Hybrid	Penicillium aethiopicum strain IBT 5753	Ascomycota (Fungi)	51400	GU574477	19168978
Compactin/mevastatin	Hybrid	Penicillium citrinum ML-236B	Ascomycota (Fungi)	38231	AB072893	12172803
Cylindrocyclophane	Hybrid	Cylindrospermum licheniforme UTEX 'B 2014'	Cyanobacteria (Bacteria)	26219	JX477167	23106426
Maduropeptin	Hybrid	Actinomadura madurae	Actinobacteria (Bacteria)	85411	AY271660	17918933
Teicoplanin	Hybrid	Actinoplanes teichomyceticus	Actinobacteria (Bacteria)	89976	AJ632270	15113000
Balhimycin	Hybrid	Amycolatopsis balhimycina DSM 5908	Actinobacteria (Bacteria)	66669	Y16952	10390204
Ristocetin	Hybrid	amycolatopsis lurida	Actinobacteria (Bacteria)	80193	KJ364518	25022591
Chloroeremomycin	Hybrid	Amycolatopsis orientalis	Actinobacteria (Bacteria)	37941	AJ223998	9545426
Difficidin	Hybrid	Bacillus amyloliquefaciens	Firmicutes (Bacteria)	69641	AJ634062	3123448
Zwittermicin A	Hybrid	Bacillus cereus strain UW85	Firmicutes (Bacteria)	65755	FJ430564	19098220
Iturin	Hybrid	Bacillus subtilis	Firmicutes (Bacteria)	41599	AB050629	11591669
Rhizoxin	Hybrid	Burkholderia rhizoxina	Proteobacteria (Bacteria)	81989	AM411073	17154220
Elansolid	Hybrid	Chitinophaga pinensis	Bacteroidetes (Bacteria)	77000	WP_012789122	21154488
Elansolid	Hybrid	Chitinophaga sancti	Bacteroidetes (Bacteria)	78240	HQ680975	21472917
Crocacin	Hybrid	Chondromyces crocatus	Proteobacteria (Bacteria)	61438	FN547928	24981773

Antibiotic name	BGC type	Microbial strain	Phylum	Length (bp)	Accession	PMID
Corallopyronin A	Hybrid	Corallococcus coralloides strain B035	Proteobacteria (Bacteria)	79373	HM071004	20503218
Barbamide	Hybrid	Lyngbya majuscula	Cyanobacteria (Bacteria)	40156	AF516145	12383521
Chondrochlorens	Hybrid	myxobacterium Chondromyces crocatus Cm c5	Proteobacteria (Bacteria)	51243	AM988861	19171307
Myxopyronin	Hybrid	Myxococcus fulvus	Proteobacteria (Bacteria)	53391	KF356280	23983106
Cryptophycin	Hybrid	Nostoc sp. ATCC 53789	Cyanobacteria (Bacteria)	57306	EF159954	17240975
Ambruticin	Hybrid	Polyangium cellulosum strain So ce10	Proteobacteria (Bacteria)	100708	DQ897667	17185223
Thiomarinol	Hybrid	Pseudoalteromonas sp. SANK 73390	Proteobacteria (Bacteria)	97600	FN689524	21483852
Mupirocin	Hybrid	Pseudomonas fluorescens NCIMB 10586	Proteobacteria (Bacteria)	75138	AF318063	12770824
Kalimantacin/batumin	Hybrid	Pseudomonas fluorescens strain BCCM_ID9359	Proteobacteria (Bacteria)	77317	GU479979	20189105
FR901464	Hybrid	Pseudomonas sp. 2663	Proteobacteria (Bacteria)	80584	HM047288	21291275
Oocydin	Hybrid	Serratia marcescens strain MSU97	Proteobacteria (Bacteria)	77781	JX315603	23012376
Oocydin	Hybrid	Serratia plymuthica strain A153	Proteobacteria (Bacteria)	78466	JX315604	23012376
Myxalamid	Hybrid	Stigmatella aurantiaca	Proteobacteria (Bacteria)	49736	AF319998	11182319
Rhizopodin	Hybrid	Stigmatella aurantiaca	Proteobacteria (Bacteria)	92218	FR854394	22278953
Kedarcidin	Hybrid	Streptoalloteichus sp. ATCC 53650	Firmicutes (Bacteria)	135420	JX679499	23633564
Napyradiomycin	Hybrid	Streptomyces aculeolatus	Actinobacteria (Bacteria)	43410	EF397638	17392281
Oxazolomycin	Hybrid	Streptomyces albus	Actinobacteria (Bacteria)	127726	EF552687	16707707
Salinomycin	Hybrid	Streptomyces albus subsp. albus	Actinobacteria (Bacteria)	94163	HE586118	22076845
Angucycline-like	Hybrid	Streptomyces ambofaciens strain ATCC 23877	Actinobacteria (Bacteria)	33064	AY338477	14742212
Indanomycin	Hybrid	Streptomyces antibioticus	Actinobacteria (Bacteria)	79604	FJ545274	19301315
Simocyclinone	Hybrid	Streptomyces antibioticus	Actinobacteria (Bacteria)	75379	AF324838	11959542
Lipomycin	Hybrid	Streptomyces aureofaciens Tü117	Actinobacteria (Bacteria)	66871	DQ176871	16723573
Neocarzinostatin	Hybrid	Streptomyces carzinostaticus subsp. neocarzinostaticus	Actinobacteria (Bacteria)	92294	AY117439	15797213
Polyketomycin	Hybrid	Streptomyces diastatochromogenes	Actinobacteria (Bacteria)	52164	FJ483966	19266534
Zorbamycin	Hybrid	Streptomyces flavoviridis	Actinobacteria (Bacteria)	84773	EU670723	19081934
C-1027	Hybrid	Streptomyces globisporus	Actinobacteria (Bacteria)	85163	AY048670	12183628
Prodigiosin	Hybrid	Streptomyces griseoviridis	Actinobacteria (Bacteria)	49027	AB469822	19329986
Griseoviridin and Viridogrisein	Hybrid	Streptomyces griseoviridis NRRL:2427	Actinobacteria (Bacteria)	123563	JX508597	23161816
Chromomycin	Hybrid	Streptomyces griseus	Actinobacteria (Bacteria)	42074	AJ578458	15112992
Vicenistatin	Hybrid	Streptomyces halstedii	Actinobacteria (Bacteria)	64492	AB086653	15112997
9-methylStreptimidone	Hybrid	Streptomyces himastatinicus	Actinobacteria (Bacteria)	52580	FR878059	4366263
Geldanamycin	Hybrid	Streptomyces hygroscopicus	Actinobacteria (Bacteria)	69644	AY179507	12586396
FK520	Hybrid	Streptomyces hygroscopicus var. ascomyceticus	Actinobacteria (Bacteria)	77534	AF235504	10863099
Streptolydigin	Hybrid	Streptomyces lydicus	Actinobacteria (Bacteria)	80894	FN433113	19875077
Enterocin	Hybrid	Streptomyces maritimus	Actinobacteria (Bacteria)	22511	AF254925	11137817
Meilingmycin	Hybrid	Streptomyces nanchangensis	Actinobacteria (Bacteria)	185250	FJ952082	12811466
Nanchangmycin	Hybrid	Streptomyces nanchangensis	Actinobacteria (Bacteria)	132544	AF521085	12770825
Desosamine	Hybrid	Streptomyces narbonensis	Actinobacteria (Bacteria)	17665	AF521878	10822548

Antibiotic name	BGC type	Microbial strain	Phylum	Length (bp)	Accession	PMID
Pimaricin	Hybrid	Streptomyces natalensis	Actinobacteria (Bacteria)	84985	AJ278573	11094342
Concanamycin A	Hybrid	Streptomyces neyagawaensis	Actinobacteria (Bacteria)	99892	DQ149987	16207901
Pactamycin	Hybrid	Streptomyces pactum	Actinobacteria (Bacteria)	42898	AB303063	17827660
Pyridomycin	Hybrid	Streptomyces pyridomyceticus strain NRRL B-2517	Actinobacteria (Bacteria)	42448	333441659	21454714
Rapamycin	Hybrid	Streptomyces rapamycinicus	Actinobacteria (Bacteria)	107379	X86780	7644502
Oxytetracycline	Hybrid	Streptomyces rimosus	Actinobacteria (Bacteria)	25222	DQ143963	16597959
Lankacidin	Hybrid	Streptomyces rochei	Actinobacteria (Bacteria)	37642	AB088224	12791134
Laidlomycin	Hybrid	Streptomyces sp. CS684	Actinobacteria (Bacteria)	102840	JQ793783	17342048
FR-008	Hybrid	Streptomyces sp. FR-008	Actinobacteria (Bacteria)	138203	AY310323	14652074
Guadinomine	Hybrid	Streptomyces sp. K01-0509	Actinobacteria (Bacteria)	51101	JX545234	23030602
Tetronomycin	Hybrid	Streptomyces sp. NRRL 11266	Actinobacteria (Bacteria)	113234	AB193609	18404760
Tirandamycin	Hybrid	Streptomyces sp. SCSIO1666	Actinobacteria (Bacteria)	55500	324330310	21329667
Liposidomycin	Hybrid	Streptomyces sp. SN-1061M	Actinobacteria (Bacteria)	29709	GU219978	20039253
Cycloheximide	Hybrid	Streptomyces sp. YIM 56141	Actinobacteria (Bacteria)	45467	JX014302	Unpublished
Tautomycin	Hybrid	Streptomyces spiroverticillatus	Actinobacteria (Bacteria)	85187	EF990140	16707708
Virginiamycin	Hybrid	Streptomyces virginiae	Actinobacteria (Bacteria)	57999	297233228	17350183
Pyrrolomycin	Hybrid	Streptomyces vitaminophilus ATCC 31673	Actinobacteria (Bacteria)	55655	EF140901	17158935

Additional table 2: Summary of NRPS and PKS gene clusters found in the genomes of bacteria from the gut

Phylum	Bacterial species	Average genome size Mb	Number of gene clusters				Average BGCs size bp
			NRPS	PKS	hybrid	total	
**Actinobacteria**	*Actinomyces grossensis*	1.69	0	1	0	1	13126
	Actinomyces ihumii	2.46	1	0	0	1	7315
	Actinomyces radingae	2.54	0	0	0	0	0
	Streptomyces massiliensis	6.53	1	3	2	6	235217
	Tessaracoccus massiliensis	3.21	0	1	0	1	7101
**Firmicutes**	*Bacillus vallismortis*	4.01	4	3	2	9	336929
	Bacillus subtilis	4.08	3	0	1	4	128496
	Bacillus ihumii	6.11	0	1	1	2	5144
	Bacillus thermoamylovorans	4.11	0	1	1	2	14112
	Bacillus andreraoultii	4.05	0	1	0	1	60847
	Bacillus badius	4.85	0	2	1	3	58327
	Bacillus idriensis	4.83	0	1	1	2	70259
	Bacillus marsiflavi	4.33	0	0	1	1	41965
	Bacillus aquimaris	4.42	0	1	0	1	15023
	Bacillus kyongensis	4.65	0	0	0	0	0
	Bacillus halodurans	3.81	0	0	0	0	0
	Bacillus jeotgali	4.63	0	0	0	0	0
	Bacillus niameyensis	4.57	0	0	0	0	0
	Bacillus oleronius	5.20	0	0	0	0	0
	Bacillus phoceensis	4.56	0	0	0	0	0
	Bacillus rubiinfantis	4.31	0	0	0	0	0
	Bacillus testis	3.97	0	0	0	0	0
	Christensenella timonensis	2.65	0	1	0	1	4382
	Clostridium aerotolerans	4.88	0	4	0	4	73836
	Clostridium mediterranense	3.05	1	1	0	2	33214
	Clostridium niameyiense	2.54	1	1	0	2	19038
	Clostridium sporogenes	4.15	1	1	0	2	29509
	Clostridium amazonitimonensis	3.75	0	1	0	1	6169
	Clostridium baratii	3.07	0	1	0	1	4755
	Clostridium bouchedurhonense	3.41	0	1	0	1	9489
	Clostridium jeddahtimonense	4.06	0	1	0	1	7312
	Clostridium neonatale	4.71	0	1	0	1	5909
	Clostridium nigeriense	3.77	0	1	0	1	4622
	Clostridium amolyticum	4.26	0	1	0	1	4632
	Enterococcus massiliensis	2.71	0	1	0	1	4766
	Murdochiella massiliensis	1.64	0	0	0	0	0
	Oceanobacillus sojae	4.79	0	3	0	3	19023
	Oceanobacillus iyehnensis	3.90	1	0	0	1	14557
	Paenibacillus barcinonensis	6.49	1	1	6	8	190824
	Paenibacillus ihumii	5.92	5	1	2	8	132411
	Paenibacillus illinoisensis	6.82	0	1	0	1	5543
	Paenibacillus numidis	5.85	0	1	1	2	31789
	Paenibacillus senegalomassiliensis	5.06	0	3	0	3	34352
	Paenibacillus touaregensis	5.66	0	2	2	4	84032
	Paenibacillus barengoltzi	5.48	0	1	0	1	4521
	Paenibacillus phoceensis	5.52	0	2	0	2	12197
	Paenibacillus rubiinfantis	5.37	0	1	0	1	3481
	Virgibacillus senegalensis	3.88	0	2	0	2	26688
**Proteobacteria**	*Pseudomonas massiliensis*	4.72	0	2	0	2	71334

Additional Figure 2: Distribution of NRPS, PKS, and hybrid gene clusters in bacteria from the gut a) in all studied bacteria, b) in the studied phyla

a.

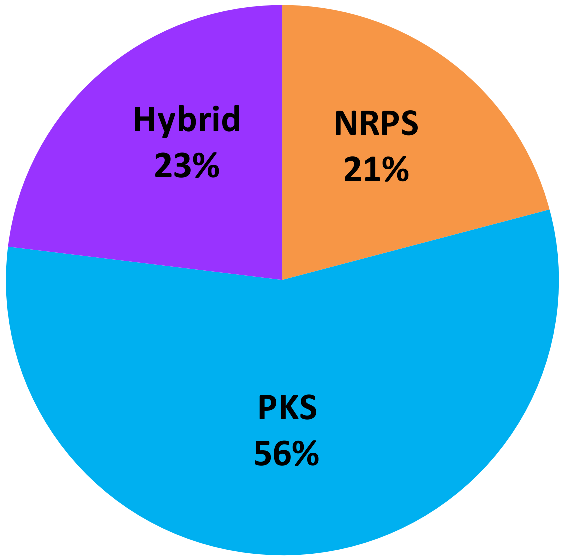

b.

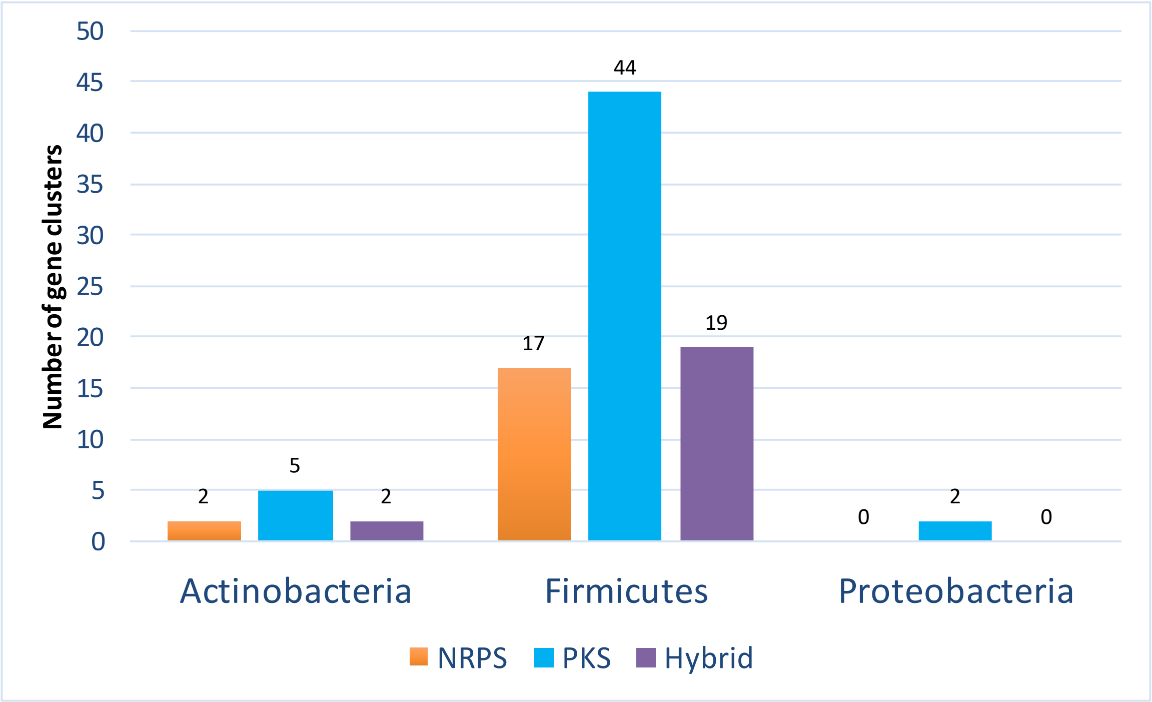

Supplement: Supplementary file 1 — Figure S1. A Venn diagram of PKS, NRPS, and hybrid gene-cluster numbers in NRPUR database. The gene-cluster numbers of the total, bacteria, eukarya and archaea are shown in black, red, green, and grey, respectively. Table S1. The 164 NRPS-PKS BGC sequences identified from the literature search resulting from experimental data with validated antibacterial activity. Table S2. Summary of NRPS and PKS gene clusters found in the genomes of bacteria from the gut. Figure S2. Distribution of NRPS, PKS, and hybrid gene clusters in bacteria from the gut a) in all studied bacteria, b) in the studied phyla (PDF 313 kb) [file 12859_2018_2479_MOESM1_ESM.pdf]
